# Quarterly Summary

**Published:** 1856-07

**Authors:** 


### QUARTERLY SUMMARY.

### DENTAL SCIENCE.

1.—*Osseous Union of Teeth.*—Mr. John J. Patrick, dentist, in the Dental News Letter for April, 1856, gives from De Loude, the particulars of a case described by M. Jasinski, in which a well developed molar tooth, with three roots, was taken from the recto-vaginal septum.*

Mr. Patrick then proceeds to state the case of a Mr. Watson, aged 50 years, who called at his office on the 27th of December, 1855, and presented his mouth for examination, calling special attention to a "twin tooth" in his lower jaw. Mr. Patrick continues to say, "upon a clear examination I found the left permanent inferior frontal and lateral incisors perfectly united from the neck up to the cutting edges. I passed a crown lance across the anterior surface of the teeth, in order to discover, if possible, a separation in the crowns, but they remained perfectly firm; the two teeth presented a plain uninterrupted enameled surface, but somewhat shorter than the adjoining teeth."†

The author of the foregoing concludes, from the circumstances connected with the development of the teeth, that such osseous union is more likely to occur in the permanent than in the temporary teeth. Observation, however, has demonstrated the converse of this opinion. Twenty examples of osseous union of the temporary to one of the permanent teeth have been met with.

2.—*Dentistry in Germany.*—Dr. F. Coas, dentist, now practicing his profession in Germany, writes to the Dental News Letter the condition of dentists and dentistry there. He presents a picture which is truly woeful—he says the file is used to a great extent, even

* Several examples of occurrences of this kind are furnished by the annals of medicine.—Eds.

† Two cases similar to the one here described are given by the Senior Editor in his "Principles and Practice of Dental Surgery," with the exception that one occurred between two central incisors in the upper jaw.—Eds.

so much as filing of one-third of a tooth away, in order to preserve the remaining two-thirds. And the filed surface being seldom polished, soon becomes blackened, which renders it still more unsightly. He remarks that filling is termed "*plumbing*," which operation is usually performed with some one of the mineral succedanea—and that he has seen whole rows of front teeth filled in this way—"presenting all the intermediate colors between chalk and charcoal."

The gold which is prepared in Germany being of such inferior quality, that he found some difficulty in making a crown filling with it. He states that a dentist takes rank with barbers and cutters of corns.

Charges for professional services of the dentist, he quotes thus: Extracting a tooth twenty kreutzers, (about 14 cents,) for "*plumbing*," from one to two florins, (40 to 80 cents,) cleaning is one and a half florins. He also says that the laws of the country forbid all foreigners from participation in trade, and mentions an instance of an unlucky wight of an Englisman who was thrown in jail for attempting to practice dentistry without permission of the authorities.

3.—*Working of Steel.*—There is published in the April No. of the Dental News Letter, a paper read by Dr. How, on this important subject. He very justly holds that every dentist should be well acquainted with the properties of steel, and the manner of working it into the several forms which are desired; and expresses some indignation that this subject should not have met with more consideration by dental writers. After which he numbers the different kinds of steel, and decides that cast steel is the best for the use of the dentist, and gives the manner in which it should be worked, so as to attain that degree of hardness most desirable.

He remarks, "that files are cut with chisel-shaped tools, struck by the hammer—and that a skillful workman will make files of different grades with the same tool. The quality of the file is graduated by the thickness, bevel and direction of the chisel, together with the weight of the blow. Experiment must teach, remarks Dr. How, the proper combination of the circumstances for the production of the required instrument."

He then proceeds to state the manner of hardening and tempering, for a cooling medium, water at ordinary temperature is best. Various mixtures are used in the manufacture of different articles.

Oil, tallow and rosin, alone or in combination, are used for articles which require a low temper; brine, mercury, acidulated and ice-water, give a greater degree of hardness than plain water, but for ordinary purposes water fulfills every necessary indication. The instrument to be hardened should be heated *slowly* and *uniformly* to a cherry-red color, and then plunged instantly into the water—if a large object, plunge vertically, and move it about in the vessel.

"Have the water close at hand, in order that no heat shall be lost during the transfer. The scale should always be removed before heating or hardening, and in the case of serrated instruments like files, the small points must be protected from oxydation, by previously coating them with yeast, thick paste, borax, or any thing which will prevent exposure to the air. Sharp angles in large objects render them liable to fracture in hardening, but in very small instruments this is not of much consequence. After hardening, brighten the surface, preparatory to drawing the temper; to do this, many ways are proposed. A simple method for small objects, is to reduce the size of the lamp-flame, by pressing in the wick, or otherwise, and hold the instrument over the flame, beginning at the base of the shank, and revolving it slowly, until the desired temper is obtained. Holding between hot pincers, resting on a bar of hot iron, plunging in a bath of fusible metal, heated to the proper temperature, and various modes of procedure, are open to choice. The object in all cases being to obtain such control over the process as shall enable the workman to fix the temper at any desired point. The steel should be rubbed quite bright, if it has not been protected during hardening, before drawing the temper, in order that the changes produced may be readily and with certainty observed; and these are marked by variations in its color, occasioned by oxydation of the surface, and the sequence is as followa: 'Straw color, yellow, dark-yellow, copper color, purple, blue, whitish-blue.' "*

4.—*Local Anæsthesia by Congelation.*—Dr. H. L. Burpee, in the April No. of the Dental Recorder, thinks that direct application of cold causes too much pain to a vital tooth or one in an inflamed condition, to be adopted into general use. He states that he has

* Elliot on Operative and Mechanical Dentistry.

adopted into his practice an apparatus with which he extracts teeth without pain, but that it requires a longer time to effect the object than the one invented by Dr. Branch, "by commencing with warm water, and graduating the cold at will, by which process the patient suffers actually nothing in the application, and seldom any pain is felt in removing the tooth." The time required is from one and a half to two and a half minutes. "This apparatus consists of a combination force pump, by which two fluids (warm and cold) can be thrown together or separately, through a flexible tube, into a mouthpiece, covering the tooth and surrounding the gum (the muscles of the face and tongue being protected by a non-conductor,) and passing off through a flexible tube leading to a vessel placed in a convenient position to receive the waste fluid." Dr. Burpee thinks that there are cases where different applications of cold may be used, to those teeth or roots which have lost their vitality. He remarks, that the danger to be incurred in anæsthesia by congelation, is as nothing compared to ether and chloroform, and that the results are quite as satisfactory, "and that it leaves none of the ill effects;" "that the patient is relieved from much dreaded pain and anxiety of mind."

### CHEMISTRY.

5.—*On the Decomposition of Iodide of Starch by the Animal Fluids.*—Dr. Dalton contributes the following paper to the *American Journal of Medical Science.*

Some months since, while experimenting on the digestion of starch, and its conversion into sugar in the intestine, I observed that the iodide of starch became very rapidly decolorized on being digested, at the temperature of 100° F., with the intestinal fluids taken from the duodenum of a recently killed dog. It appeared, also that this effect was produced some time before any sugar had made its appearance in the mixture, and would even take place, with nearly equal promptness, at the ordinary temperature of the air. It seemed, at first, probable, that the decolorization was owing to a partial alteration of the starch, which passed into an intermediate condition, before undergoing its final conversion into sugar. Subsequent examination, however, showed that it was due to a simple decomposition of the iodide of starch by the organic substances of the intestinal fluids. Nearly all the animal fluids, indeed, when in a fresh condition, not only discolorize, more or less promptly, the blue iodide of starch, but prevent entirely the usual reaction between starch and iodine when this latter substance is present in small quantity. When the iodine of starch, ready formed, comes in contact with these fluids, the iodine leaves the starch and combines with the organic matters; and it is under the form of such an organic combination that the iodine is absorbed from the intestine, passes through the circulation, and is discharged by the urine. The precaution, therefore, which is sometimes inculcated, in the administration of free iodine, to give the medicine in the interval between two meals, lest it should combine with the starchy matters of the food, and become insoluble and inert, is altogether superfluous; since starch cannot retain the iodine in combination with it, in presence of the digestive fluids.

The iodide of starch, in the form of a paste, has, indeed, been occasionally recommended as one mode of administering the drug; but I am not aware that the mode in which iodine, in this state of combination, becomes absorbed, has yet been directly investigated.

The solution of starch employed in the following experiments, was made by boiling five grains of starch in the ounce of water, and allowing the fluid to cool. The experiments were all made at the ordinary temperature of the atmosphere.

1. If ʒ i of *saliva* be mixed with ʒ i of iodine water, and ʒ ss of the starch solution added, no blue color is produced; but a drop or two of nitric acid immediately turns the mixture blue and opaque.

ʒ i of iodine water is added to ʒ i of the starch solution, making an opaque blue fluid; ʒ ii of saliva are then added, and the whole shaken up. The mixture becomes nearly colorless at the end of five minutes. The blue color is then restored by a drop or two of nitric acid.

2. If ʒ i of iodine water, with ʒ i of starch, be mixed with an equal quantity of fresh *pancreatic juice* (from the dog,) the blue color is entirely dissipated in less than one minute.

3. Light yellow, nearly clear *bile*, from the dog's gall-bladder, destroys immediately the blue color of an equal quantity of starch and iodine water. On the addition of nitric acid, the blue color is restored, modified somewhat by the green tinge which the bile takes with nitric acid.

4. A healthy dog, kept for 24 hours without food, was killed by section of medulla, and the scanty, light yellowish, frothy *fluid of the small intestine* collected. It discolorized immediately the blue iodide of starch, made in the above manner, and the blue color was restored by nitric acid.

A dog was kept for 24 hours without food, then fed with fresh lean meat, and killed half an hour after feeding. The intestinal fluids from the upper half of the small intestine acted on the iodide of starch in the same manner as in the last experiment.

5. If ʒ ij of the clear yellowish *serum of the blood* (horse's blood) be added to ʒ ij of iodide of starch, the mixture loses its blue color immediately. It is brought back by nitric acid.

6. If ʒ i of healthy human *urine* be added to ʒ i of iodide of starch, the mixture is decolorized in five seconds. The blue color is restored by nitric acid.

The above effect is produced whether the urine be acid or slightly alkaline in reaction.

ʒ i of urine mixed with ʒ v of iodine water effectually prevents the reaction between the iodine and starch.

7. The *gastric juice* acts differently, in this respect, according to circumstances. If taken from the stomach of the fasting animal (by irritation, through a gastric fistula, with a metallic catheter,) it is clear and colorless, and does not interfere at all with the reaction of starch and iodine. But that which is collected during the first half hour after feeding the animal on cooked meat, and which is slightly yellowish in color, and contains a little albuminose in solution, interferes with the reaction very perfectly. ʒ i of such gastric juice decolorizes an equal quantity of iodide of starch in less than a minute.

A specimen of such gastric juice, however, containing a little albuminose, which had been kept for some months, was found to have lost almost entirely its power of preventing the union between starch aud iodine.

If the colorless gastric juice, taken from the fasting animal, which has no effect on the iodide of starch, be digested for an hour at the temperature of 100° F., with finely cut boiled meat, and then filtered, the filtered fluid interferes with the reaction of iodine and starch, like the other animal fluids.

The iodine, in these cases, probably combines, as already intimated, directly with the organic (albuminoid) matters of the animal fluids, and is detected by starch only after these organic matters have been destroyed by nitric acid. It is not, however, easy to demonstrate this. If the serum of the blood, for example, be boiled with an excess of sulphate of soda, by which all of the albumen, and most of the other organic substances are coagulated, the clear filtered fluid interferes with the reaction of starch and iodine as before. It might be supposed that the iodine, on the contrary, was converted into an iodide of potassium or sodium, under the influence of the alkaline carbonates, and that it passed, under this form, through the circulation and into the excretions. Several circumstances, however, are opposed to this view. First, the iodide of starch is decomposed, not only by the saliva, serum of the blood, and pancreatic juice, which are alkaline; but also, quite as promptly, by the urine and gastric juice, which are acid in reaction. Secondly, the gastric juice, as already mentioned, has no power of preventing the union of starch and iodine, when taken fresh from the stomach of the fasting animal, but yet gains this power in a high degree when artificially digested with meat at 100° F. It seems nearly certain, therefore, that the iodine combines, in this instance, with some of the albuminoid matters dissolved by the gastric juice.

This reaction of iodine with the animal substances is further illustrated, in a very striking manner, by the following experiments, performed at the suggestion and with the assistance of my friend, Dr. Wm. H. Ellett:

I. If a mixed solution of boiled starch and iodide of potassium be spread upon the surface of the skin, and the two poles of a galvanic battery applied to the moistened surface a short distance from each other, no perceptible discoloration appears, provided the galvanic current be not very powerful; while if the same poles be applied to a piece of bibulous paper, moistened with the same solution, the blue iodide of starch appears at once at the positive pole.

II. If the negative pole of the battery be placed in contact with the bibulous paper, moistened as above, the positive pole taken in the left hand, and the circuit completed by touching the bibulous paper with the right forefinger, the shock is felt, but no discoloration of the paper is produced; but if the circuit be completed by a steel needle, held in the right hand, a dark stain immediately appears on the paper.

III. The negative pole was placed in contact with the moistened paper, and a fresh piece of animal membrane (uterus of the cow) attached by one end to the positive pole. The other end of the animal membrane was then cut to a fine point, by which the circuit was completed on the paper; but no stain occurred. A steel needle was then thrust through the animal membrane, and the circuit completed by the point of the needle, when the discoloration of the paper immediately appeared.

It appears, then, that whenever iodine is set free from its combination with potassium, in presence of starch and an animal organic substance, either living or recently dead, it combines with the organic substance in preference to uniting with the starch.

The influence of the animal fluids in preventing the mutual reaction of iodine and starch is, as might be expected, not unlimited, but depends on the relative quantities of the iodine and the organic substance with which it combines. If there be but little iodine present, it is all taken up by the organic substance, and shows no reaction with starch; while, if present in large quantity, it attacks the starch also, and produces the characteristic blue color. Thus, one drachm of iodine water, mixed with one drachm of gastric juice holding albuminose in solution, has no reaction on starch; but a mixture of two drachms of the former to one drachm of the latter, strikes with starch a perceptible blue color. A single drop of the alcoholic tincture of iodine, mixed with one drachm of gastric juice, is sufficient to produce a blue color with one drop of the starch solution. Other animal fluids, however, appropriate larger quantities of free iodine. Two drops of tincture of iodine, for example, are completely saturated by one drachm of healthy urine, so that the mixture gives no reaction with starch; three drops give only a purplish color, while four drops strike a deep blue color on the addition of starch.

With the adoption of proper precautions, the reactions described above will not expose us to any serious error when endeavoring to ascertain the presence of either starch or iodine in the animal fluids. In testing for iodine, we have only to add a little nitric acid after dropping in the starch; and in testing for starch, we should be sure to add iodine enough to more than saturate the albuminoid substances present. It is at least evident, however, from the foregoing facts, that iodine cannot possibly exist in a free state in the animal fluids, but is always in either a metallic or an organic combination.

6.—*Physiology of the Spinal Cord.*—The prize of experimental physiology, for the year 1855, has been awarded by the French

Academy of Sciences to M. Brown-Sequard. This award was unanimously recommended by the distinguished committee, consisting of MM. Flourens, Serres, Rayer, Magendie, and Cl. Bernard, to which the memoir of M. Brown-Séquard was referred. The following report, drawn up M. Cl. Bernard, gives the fullest account we have met with of the results of M. Brown-Séquard's researches:

It was suspected, at a very early period, by physiologists and physicians, that the different vital phenomena due to the nervous system, and especially, those of sensibility and motion, had distinct organs for their transmission. It is to modern physiology, which has carried so far experimental analysis into the functions of the nerves, that the credit is due of having made the important discovery, and established, by incontrovertible evidence, that the anterior roots of the spinal marrow are the motor nerves, and the posterior roots the nerves of sensation. Or, in other words, that when a voluntary movement takes place, in one of the limbs for example, the motive influence originating in the encephalic centre of the spinal marrow can be transmitted to the proper muscles only by the anterior spinal roots; and that when a sensitive impression is conveyed in the opposite direction, from the periphery of the body to the nervous centres, this can be transmitted to the spinal marrow, and from thence to the encephalon, only by the posterior spinal roots.

While all physiologists are now agreed as to the manner in which the motive and sensitive functions are localized in respect to the spinal nerves, it is not so as to the spinal marrow itself. Are sensation and movement propagated within the spinal marrow by distinct portions of the cord? And if so, which are the portions which transmit the motive influence and which the sensitive impressions? The solution of these important questions has been attempted by the most skillful experimenters, but they still remain undecided. On the one hand, it is contended that the white matter of the cord is unadapted to transmit either sensation or motion, and that the gray matter is alone endowed with this double function, or partakes of it with the white portion. On the other hand, it is maintained that the white matter of the cord is the sole conducting medium, it being supposed, as an established fact, that the posterior fasciculi, which are in connection with the posterior spinal roots, are the exclusive conductors of sensitive impressions, while the antero-lateral fasciculi which are contiguous to the anterior spinal roots are the organs for the transmission of motion. And it may be remarked, this last opinion is that which has been most generally adopted, at least in France.

M. Brown-Séquard has recently examined anew this difficult question of the transmission of sensitive impressions and motion in the spinal marrow, and the better to limit his subject, he has divided the problem into two; occupying himself, at first, with the determination of the parts of the cord by which sensitive impressions are transmitted from the posterior roots to the encephalic centre. It is, therefore, exclusively to the transmission of sensitive impressions in the spinal marrow that the present researches of M. Brown-Séquard, as well as the experiments performed by that learned physiologist before the commission are confined.

The first proposition that he would establish, is that the posterior fasciculi of the spinal marrow are not, as has been asserted, the exclusive agents for the transmission of sensitive impressions. To prove this, M. Brown-Séquard has performed two leading experiments.

The first experiment consists in cutting through, in a living animal, the posterior fasciculi of the spinal marrow on a level with the dorsal region. When, after this division, we pinch the posterior limbs of the animal, it feels it perfectly, and manifests immediately by its cries the pain it experiences. This result shows evidently that the posterior fasciculi of the cord are not exclusively charged with the transmission of sensibility, inasmuch as the sensitive or painful impression excited in the posterior limbs is transmitted to the brain, after the complete division of the fasciculi above the origin of the nerves of the posterior limbs, and consequently, at a point between the nerve, the pinching of which has caused the sensation of pain, and the brain where that sensation arrives to be appreciated.

Another most interesting phenomenon which has been discovered by M. Brown-Séquard, is that if, in this experiment, we pinch or irritate the posterior fasciculi of the cord at the part where they have been divided, we not only perceive that the two divided surfaces of the fasciculi are sensible, but we usually observe that the inferior or caudal end is more sensible than the superior or cephalic end, which alone remains in direct continuity with the encephalon.

It is unnecessary to remark that this new fact is, also, in opposition to the theory of the exclusive transmission of sensibility by the posterior fasciculi. According to this theory, the same effect should occur after the division of the posterior fasciculi, as after the section of the posterior spinal roots—namely, the end which remains in direct continuity with the encephalic centre should alone remain sensible, while the peripheral end should become completely insensible.

The second experiment of M. Brown-Séquard is in some degree the counter proof of the first.

We have just seen that the posterior fasciculi of the cord have been divided, in order to show that, without their intervention, sensitive impressions may be transmitted to the brain by other portions of the spinal marrow that remain intact. It can still further be shown, that by the posterior fasciculi alone, when the other portions of the spinal marrow have been divided, the transmission of sensitive impressions cannot take place. This experiment has been performed by M. Brown-Séquard. He divided, upon a living animal, on a level with nearly the second dorsal vertebra, all the spinal marrow, with the exception of the posterior fasciculi. Immediately after this division was made, the posterior limbs of the animal became completely paralyzed, and when they were pinched no sensation was excited; that is to say, the transmission of sensitive impressions did not take place, notwithstanding the posterior fasciculi remained intact.

These two experiments, therefore, logically enchain themselves to prove that the posterior fasciculi are not the organs by which sensitive impressions are transmitted in the spinal marrow. The experiments were repeated before the commission by M. Brown-Séquard, with great skill, upon animals in which the spinal cord was not exposed but for a very limited extent, and so as not to cause debility from hemorrhage, and to obtain the most conclusive results.

M. Brown-Séquard in his memoir examines afterwards the rôle of the gray substance, as well as the anterior and lateral cords of the spinal marrow, relative to the transmission of sensitive impressions. He has performed a number of experiments, in which he has treated the anterior and lateral cords in the same manner as he had the posterior cords, and has arrived at precisely analogous results. M. Brown-Séquard has seen, in effect, that after the section of the posterior cords, of the lateral cords, and of the anterior cords of the spinal marrow, sensitive impressions can yet be experienced, while, when the gray matter is destroyed, the transmission of sensitive im-

41*

pressions immediately ceases, even when the greater portion of the medullary fasciculi is allowed to remain as much as possible undisturbed. By all these numerous researches, M. Brown-Séquard has been led to the conclusion, that no portion of the white matter of the spinal marrow possesses the function of transmitting sensitive impressions to the centre of perception, but that this transition is effected by the gray medullary matter, especially in central portion. These results are of great interest in the physiology of the nervous centres, inasmuch as they show that insensible parts, as the gray matter of the spinal cord, are able to transmit sensitive impressions, while parts endowed with great sensibility, as the posterior fasciculi of the cord, do not transmit them.

In the second part of his memoir, M. Brown-Séquard has endeavored to determine, experimentally, how the sensitive fibres of the posterior spinal roots, which convey sensitive impressions from the periphery, penetrate the spinal marrow in order to reach the gray matter. Supporting himself, on the one hand, upon the microscopic anatomy of the cord, and, on the other hand, upon ingeniously devised physiological experiments, he has been led to the enunciation of new views, in respect to the manner in which sensations are propagated to the central gray matter of the spinal marrow—which views he has detailed in his memoir, and which prove that this phenomenon is more complicated than we should be disposed to consider it on first view.

To conclude, the experiments of M. Brown-Séquard have thrown light upon one of the most important and difficult questions connected with the physiology of the spinal marrow—that relative to the transmission of sensitive impression by this portion of the cerebro-spinal axis. If some facts in relation to the subject were already known, M. Brown-Séquard has added to these many additional ones. He has varied his experiments, and has so arranged the results of these as to resolve, in a very satisfactory manner, the question which was the subject of his researches. The commission have consequently unanimously awarded to him the prize for experimental physiology, for the year 1855.—*Moniteur des Hôpitaux*, Feb. 5, 1856.

